# Competition of Candida glabrata against Lactobacillus is Hog1 dependent

**DOI:** 10.1111/cmi.12943

**Published:** 2018-09-07

**Authors:** Reinhard Beyer, Zeljkica Jandric, Christoph Zutz, Christa Gregori, Birgit Willinger, Ilse D. Jacobsen, Pavel Kovarik, Joseph Strauss, Christoph Schüller

**Affiliations:** ^1^ Department of Applied Genetics and Cell Biology (DAGZ) University of Natural Resources and Life Sciences, Vienna (BOKU) Tulln Austria; ^2^ Department of Farm Animal and Veterinary Public Health Institute of Milk Hygiene, Milk Technology and Food Science Vienna Austria; ^3^ Platform Bioactive Microbial Metabolites (BiMM); ^4^ Division of Clinical Microbiology, Department of Laboratory Medicine Medical University of Vienna Vienna Austria; ^5^ Research Group Microbial Immunology, Leibniz Institute for Natural Product Research and Infection Biology Hans‐Knöll‐Institute (HKI) Jena Germany; ^6^ Max F. Perutz Laboratories University of Vienna, Vienna Biocenter (VBC) Vienna Austria

## Abstract

*Candida glabrata* is a common human fungal commensal and opportunistic pathogen. This fungus shows remarkable resilience as it can form recalcitrant biofilms on indwelling catheters, has intrinsic resistance against azole antifungals, and is causing vulvovaginal candidiasis. As a nosocomial pathogen, it can cause life‐threatening bloodstream infections in immune‐compromised patients. Here, we investigate the potential role of the high osmolarity glycerol response (HOG) MAP kinase pathway for C. glabrata virulence. The C. glabrata MAP kinase CgHog1 becomes activated by a variety of environmental stress conditions such as osmotic stress, low pH, and carboxylic acids and subsequently accumulates in the nucleus. We found that CgHog1 allows C. glabrata to persist within murine macrophages, but it is not required for systemic infection in a mouse model. *C. glabrata* and Lactobacilli co‐colonise mucosal surfaces. Lactic acid at a concentration produced by vaginal *Lactobacillus* spp. causes CgHog1 phosphorylation and accumulation in the nucleus. In addition, CgHog1 enables C. glabrata to tolerate different *Lactobacillus* spp. and their metabolites when grown in co‐culture. Using a phenotypic diverse set of clinical C. glabrata isolates, we find that the HOG pathway is likely the main quantitative determinant of lactic acid stress resistance. Taken together, our data indicate that CgHog1 has an important role in the confrontation of C. glabrata with the common vaginal flora.

## 1 INTRODUCTION

Superficial and systemic infections with *Candida* species afflict millions of people worldwide. It is estimated that each year, 1.5 to 2 million people worldwide die of a fungal infection (Denning et al., 2015) and that about 10% of all bloodstream infections in the United States are caused by fungi (Wisplinghoff et al., 2004). Several *Candida* species are intimately connected to humans as they are a part of the human microbiome and appear both as commensals (e.g., in the gut) and pathogens (Huffnagle et al., 2013). They can cause life‐threatening bloodstream infections especially in immune‐compromised patients, such as preterm neonates and patients undergoing prolonged treatment in an intensive care unit (Sardi et al., 2013). More commonly, *Candida* spp. cause mucosal infections such as vulvovaginal candidiasis (VVC) causing significant discomfort and inconvenience for the afflicted (Sobel, 2007, 2016). In the northern hemisphere, *Candida albicans* and *Candida glabrata* are the two most common species isolated from VVC patients. C. glabrata belongs to the Nakaseomyces group, which includes other pathogenic yeasts and is phylogenetically related to Saccharomyces cerevisiae (Gabaldon et al., 2013). The intrinsic resistance of C. glabrata to fluconazole (and occasional resistance to echinocandins) advanced C. glabrata to the second most isolated *Candida* spp. and, due to the few available classes of antifungal drugs, renders effective treatment difficult (Rodrigues et al., 2014, Sardi et al., 2013, Silva et al., 2012). VVC often coincides with increased pH due to a dysbiosis of the vaginal microbiome, allowing *Candida* growth (Green et al., 2015). The native bacterial flora, which is often dominated by *Lactobacillus* spp., maintains a low pH and exerts a fungistatic effect against *Candida* spp. (Parolin et al., 2015, Chew et al., 2015, Hutt et al., 2016). In addition, certain *Lactobacillus* spp. are also known to enhance recruitment of phagocytic cells and propagate efficient release of pro‐inflammatory cytokines (Villena et al., 2011). Restoring the vaginal microbiome with *Lactobacillus* spp. is suggested as safe alternative treatment in case of antifungal‐resistant *Candida* spp. (Van Kessel et al., 2003, Villena et al., 2011).

C. glabrata has a highly malleable genome (Polakova et al., 2009) with an expanded family of *EPA* genes facilitating adherence, some of them not shared with S. cerevisiae (Cormack et al., 1999, Desai et al., 2011). Within the host, C. glabrata survives by modulating the content of the phagolysosome and preventing phagosome maturation, enabling it to escape (reviewed in Kasper et al., 2015). C. glabrata is taken up by macrophages more efficiently than C. albicans but adapts to the situation, reproduces within the macrophage, and then gets released after the macrophage bursts (Seider et al., 2011). Adaptable resistance to abiotic stress is an important determinant for the competitiveness of any microorganism, especially if it encounters fluctuating environments. In fungi, the high osmolarity glycerol (HOG) pathway is activated in response to osmotic stress caused by high extracellular salt or sugar concentrations. This pathway has been explored in great detail in S. cerevisiae, where it mainly reacts to osmotic stress (for review, see Saito et al., 2012, Brewster et al., 2014), but also to other stress signals (Mollapour et al., 2006, Lawrence et al., 2004, Bilsland et al., 2004, Rodriguez‐Pena et al., 2010). Hog1 is the MAPK of the HOG signalling pathway and upon phosphorylation relays information to many different targets both in the cytosol and the nucleus to increase the intracellular glycerol level to preserve an optimal turgor pressure. Deletion of *HOG1* leads to attenuated virulence, for example, in C. albicans (Alonso‐Monge et al., 1999), Cryptococcus neoformans (Bahn et al., 2005) and C. glabrata (Srivastava et al., 2015).

Interestingly, despite the high degree of conservation, regulation of the HOG pathway differs between species. In *Schizosaccharomyces pombe*, the orthologous Spc1 MAPK pathway is not only activated by osmotic stress but also by a wide range of environmental stresses such as UV radiation and heat stress (Toone et al., 1998, Chen et al., 2003). Furthermore, C. glabrata CgHog1 is activated by weak organic acid stress (Jandric et al., 2013, Gregori et al., 2007). In C. albicans, the transcriptional response to osmotic stress and heavy metal stress is partly Hog1 dependent (Enjalbert et al., 2006). C. albicans Ca*hog1*Δ mutants are sensitive against oxidative stress (Brown et al., 2009, Correia et al., 2016, Alonso‐Monge et al., 2003), but CaHog1 appears to be dispensable for induction of oxidative stress resistance. Therefore, the signals relayed by the HOG pathway differ even among closely related organisms.

The HOG pathway can influence a host–pathogen relationship in many ways as it is at the crossroad of many mechanisms involved in host–pathogen interactions. Here, we investigate the role of CgHog1 under different environmental conditions and to explore its involvement in virulence‐associated traits. We found that C. glabrata CgHog1 protects against the secreted metabolites of *Lactobacillus* spp. and allows it to compete with co‐colonising *Lactobacillus* spp.

## 2 RESULTS

### 2.1 CgHog1 protects C. glabrata from growth inhibition caused by weak organic acids and low pH

**Figure 1:**
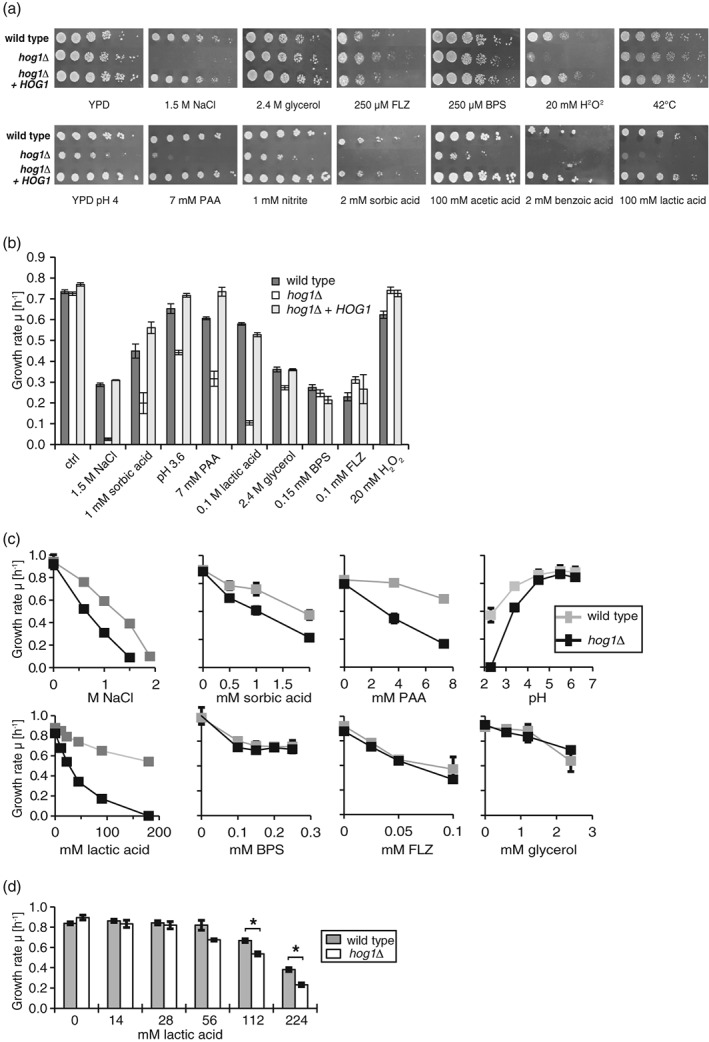
A Cghog1Δ strain is sensitive of to weak acids and low pH. (a) Growth inhibition tests of wild type and Cg*hog1*Δ strains. Stationary wild type (BG14) and mutant (BG14 *hog1*Δ) cells were spotted in serial dilutions ranging from OD_600nm_ of 1 to 3 × 10^−4^ onto YPD plates (upper panel) or YPD pH 4 plates (lower panel) containing the indicated concentrations of the respective stress agent. Plates were incubated at 37°C for 24 hr. The Cg*hog1*Δ mutant showed impaired growth on NaCl, low pH, and organic acids and a slight sensitivity towards H_2_O_2_ and nitrite. Growth of the mutant was unaffected by glycerol, iron starvation (BPS), fluconazole (FLZ), or heat stress (42°C). (b‐d): Growth experiments were run at least in triplicate with three biological replicates on the same plate. Maximal growth rate μ [h^−1^] was calculated with GROFIT. BG14 wild type, BG14 *hog1*Δ, and the BG14 *hog1*Δ*HOG1* strains were cultivated in YPD or under indicated stress conditions in 96‐well format for 24 hr at 37°C. (b) Different stress conditions reveal sensitivity phenotypes of the Cg*hog1*Δ mutant. Significant sensitivity was observed for 1.5 M of NaCl (*p* < 0.0001), 2 mM of sorbic acid (*p* < 0.01), 7 mM of PAA (*p* < 0.0001), pH 3.6 (*p* < 0.01), and 100 mM of lactic acid (*p* < 0.0001). (c) A range of concentrations as indicated was tested for each stress condition. (d) Growth rates of BG14 wild type and *hog*1Δ when grown in YPD containing lactic acid adjusted to pH 7. Asterisks indicate a significant difference between wild type and Cg*hog1*Δ (*p* value <0.001)

Here, we explored the role of CgHog1 in response to alternative stress types. Unlike in S. cerevisiae, C. glabrata Hog1 confers resistance not only to osmotic stress and sorbic acid stress but also to other stress types (Jandric et al., 2013, Srivastava et al., 2015, Gregori et al., 2007). To cover monocarboxylic acids, we chose lactic acid and PAA in addition to sorbic acid as model substrates. Lactic acid commonly occurs as a by‐product of *Lactobacillus* spp. metabolism. PAA and the derivatives 3‐phenyl lactic acid, 3‐indole acetic acid, and indole‐3‐carboxylic acid have fungicidal activity. Wild type, Cg*hog1*Δ and a reconstituted Cg*hog1*Δ mutant (Cg*hog1*Δ*HOG1*) were spotted on agar containing the selected compounds as well as a number of other stress agents. Tests with acids and nitrite were performed at pH 4, whereas all other substances were tested at neutral pH. Low pH causes a slight growth inhibition of the Cg*hog1*Δ strain and weak organic acids further enhance this effect (Figure 1a). The mutant strain also shows a slight inhibition when challenged with H_2_O_2_ or NO_2_^−^. Interestingly, osmotic stress caused by 2.4 M of glycerol had no effect on the Cg*hog1*Δ mutant, despite the similar osmotic strength to 1.5 M of NaCl (3 osmol/L of NaCl vs. 2.4 osmol/L of glycerol).

To measure subtle phenotypes, which might have escaped the qualitative analysis of spotting assays, we quantified growth parameters, such as the peak growth rate μ_max_ [h^−1^] during the exponential phase of growth, the lag phase [h] or the maximum growth level [AU]. Results of wild type and Cg*hog1*Δ strains are shown in Figure 1b. Compared with the wild type, Cg*hog1*Δ showed significantly reduced growth rates when exposed to osmotic stress, organic acids, and low pH. Some of the conditions tested caused other parameters of the medium to change as well. The effect of the low pH was subtracted by preparing a separate control sample adjusted to pH 4 using HCl when evaluating the effect of nitrite, sorbic acid, PAA, and lactic acid, which were all performed at pH 4. We calculated the final growth rate as μ_final_ = μ_measured_ * μ_YPD_/μ_Ctrl_, where μ_measured_ is the initial noncorrected growth rate, μ_YPD_ is the growth rate obtained in YPD, and μ_Ctrl_ is the growth rate obtained in the separate control assay, with adjusted pH or osmolarity. With these corrections, we calculated the CgHog1‐dependent resistance not only to sorbic acid but also to the other weak acids lactic acid and PAA as well as to pH 3.6 (Figure 1b). For glycerol, no sensitivity phenotype was observed, despite its osmotic properties. High concentrations of NaCl inhibited growth in both the mutant and the wild type, whereas equimolar amounts of glycerol did not (Figure S1A). We did not observe any hypersensitivity of the mutant to fluconazole, iron starvation, and high temperature and prolonged desiccation (Figure 1b, Figure S1B).

Interestingly, oxidative and nitrosative stress did not result in reduced growth rate (μ_max_) in the mutant, as our previous results with drop tests on solid medium would suggest (Figure 1a). It appears, however, that oxidative and nitrosative stress both lead to an increased lag phase (Figure S1C). This suggests a reduced ability of the mutant to adapt to the nitrosative and oxidative stress. In addition, we tested a wide range of concentrations and pH values and observed that the Cg*hog1Δ* strain was hypersensitive to any tested concentration of sorbic acid (0.5–2 mM), PAA (4–7 mM), and lactic acid (11–180 mM) and to low pH when compared with the wild type (Figure 1c). Results obtained from weak acid experiments were corrected for the low pH effect. Cg*hog1Δ* was also sensitive to lactic acid at high concentrations at neutral pH, (Figure 1d). Therefore, Cg*hog1*Δ mutants are hypersensitive to physiological concentrations (Owen et al., 1999) of lactic acid and other weak organic acids.

### 2.2 CgHog1 in vitro protects C. glabrata from co‐cultured *Lactobacillus* spp

**Figure 2:**
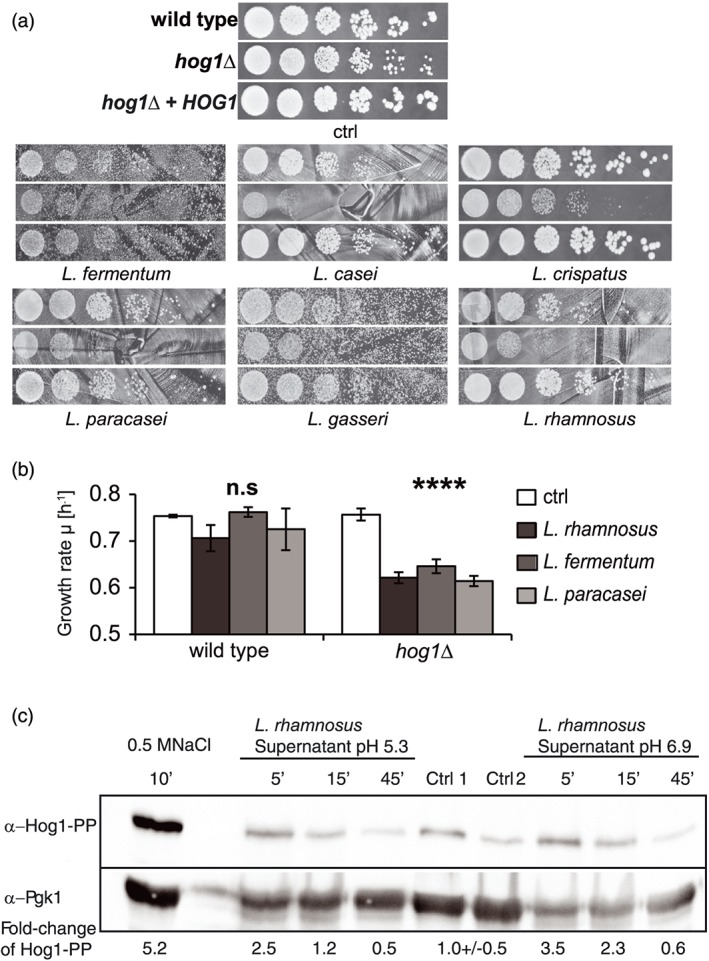
*Candida glabrata hog1∆* is inhibited by *Lactobacillus* spp. supernatant and in co‐culture. (a) Growth tests of BG14 wild type and *hog1*Δ strains on a pre‐grown lawn of *Lactobacillus* spp. on MRS plates. Exponentially growing wild type (BG14), mutant (BG14 *hog1*Δ), and revertant (BG14 *hog1*Δ + *HOG1)* cells were spotted in serial dilutions ranging from OD_600nm_ of 1 to 3 × 10^−4^ onto the bacterial lawn and incubated overnight at 37°C. (b) BG14 wild type and Cg*hog1*Δ strains were grown in supernatant of stationary *Lactobacillus* spp. cultures. Supernatants containing 6–8 g/L of lactic acid were replenished with fresh nutrients and neutralised to pH ~6.5. The Cg*hog1*Δ mutant shows significantly decreased growth rates in the bacterial supernatant compared with the control. (*p* values: *Lactobacillus rhamnosus*: 0.00019, *Lactobacillus fermentum*: 0.00069, *Lactobacillus paracasei*: 0.00022). (c) L. rhamnosus culture supernatant transiently induces CgHog1 phosphorylation. Western blot using α‐Hog1‐PP antibodies to detect the double phosphorylated form of CgHog1 in C. glabrata strain BG14 when challenged with supernatants from a L. rhamnosus strain. Pgk1 was used as loading control. Fold‐changes of Hog1 phosphorylation signal relative to a nontreated control in YPD and osmotic stress (0.5 M of NaCl) are indicated. L. rhamnosus supernatant was supplemented with YPD and added directly (pH 5.3) or adjusted to neutral pH 6.9

*Lactobacillus* spp. are a common part of the vaginal microbiome. They create a first line of defence against potentially pathogenic, microorganisms by production of stress factors, for example, organic acids. We tested the role of CgHog1 in the *C. glabrata–Lactobacillus* spp. competition. Indeed, we observed impaired growth of the Cg*hog1*Δ mutant when directly challenged with six different clinical isolates of *Lactobacillus* spp. (L. rhamnosus, *L. gasseri*, L. paracasei, L. fermentum, *L. casei*, and L. crispatus) in a co‐cultivation on MRS agar plates. In comparison, the wild type strain was less affected (Figure 2a). To exclude a pure pH effect, we challenged C. glabrata with pH‐adjusted supernatants from three of the *Lactobacillus* spp. and still observed growth inhibition of the Cg*hog1*Δ mutant (Figure 2b). This effect also occurred in heat‐ or protease‐treated supernatants (data not shown), thus excluding a polypeptide as a possible cause. We investigated the activity of CgHog1 upon exposure to culture supernatants obtained from L. rhamnosus. As shown in Figure 2c, the level of double phosphorylated Hog1 increased immediately (5 min) after the challenge, but remained lower than the osmotic stress control treatment. Additionally, the response of Hog1 rapidly diminished after 15 min and decreased even below the basal level of untreated cells. Adjusting the supernatant to a neutral pH did not change the activation kinetics of Hog1, and thus, the culture pH does not contribute to the observed transient activation. The combination of low pH and lactic acid is therefore a major contributor to inhibition *of C. glabrata* by *Lactobacillus* spp., and the HOG pathway is contributing to the equilibrium between these species in co‐culture.

### 2.3 CgHog1 localises into the nucleus following treatment with low pH stress or lactic acid

**Figure 3:**
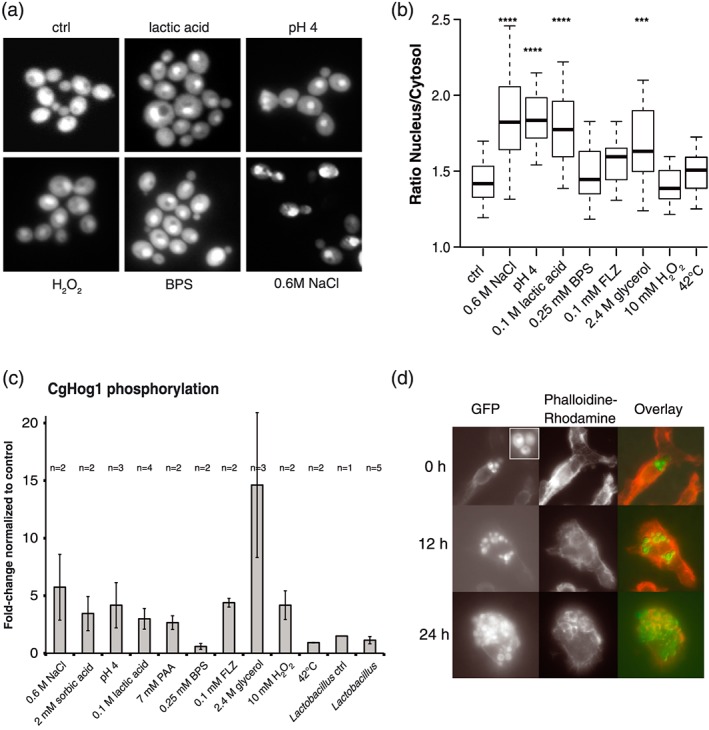
CgHog1‐GFP relocates to the nucleus in response to several stress conditions (a). Representative live microscopy pictures of BG14 *hog1*Δ expressing CgHog1‐GFP without stress or under stress conditions. (b) Nuclear accumulation of Hog1‐GFP upon stress induction results in a higher nucleus to cytosol signal ratio. Fluorescence intensity ratios (nucleus to cytosol) of cells grown exposed to indicated conditions are given (*n* ≥ 40). Compared with the nonstressed control, exposure to NaCl, low pH 4, lactic acid, and glycerol significantly increased nuclear accumulation of Hog1‐GFP. FLZ = fluconazole. The Kruskal–Wallis test was used to test for significance (***p < 0.001; ****p < 0.0001). (c) Levels of dually phosphorylated CgHog1 detected in BG14 wild type when exposed to different stress agents. CgHog1 was detected using a polyclonal anti‐phospho‐p38 MAPK antibody. All values were referenced to an untreated control sample. Treatments with NaCl, weak acids, low pH, glycerol, fluconazole, and H_2_O_2_ led to increased phosphorylation levels, whereas iron starvation (BPS), heat stress (42°C), and exposure to *Lactobacillus* supernatant do not activate CgHog1. (d) Macrophage engulfment causes transient nuclear accumulation of CgHog1‐GFP. Cells were incubated at 37°C with macrophages in a 4:1 ratio. For microscopy, cells were fixed at indicated time points and stained with Phalloidin Texas‐Red. Fluorescence microscopy shows accumulation of CgHog1‐GFP in the nucleus directly after phagocytosis and proliferation of *Candida glabrata* within the phagolysosome after engulfment after 12 hr and 24 hr

Activation of the HOG pathway is leading to Hog1 phosphorylation and subsequent nuclear accumulation. A fully complementing GFP‐tagged CgHog1 (Jandric et al., 2013) was used for microscopy to determine the ratio of nuclear versus cytosolic fluorescence (N/C‐ratio) (Figure 3a). As reference for nuclear accumulation of CgHog1, 0.6 M of NaCl was used, which showed an N/C ratio of 1.9 (*p* < 0.0001), whereas the untreated control had an N/C ratio of 1.4. Increased localisation of CgHog1 was observed for treatment with 100 mM of lactic acid, 2.4 M of glycerol (*p* ≤0.0001) and low pH (Figure 3b). Treatments that did not inhibit growth of Cg*hog1*Δ failed to enhance nuclear localisation of CgHog1 (i.e., 250 μM of BPS, 32 μg/ml of fluconazole, 20 mM of H_2_O_2_ or 42°C). However, glycerol treatment caused significant accumulation of CgHog1‐GFP in the nucleus, although no growth phenotype was observed previously. Activated CgHog1 isoforms were detected by immunoblotting using phospho‐specific antibodies recognising double phosphorylation at T180 and Y182 (CgHog1‐PP). As positive control, cells were challenged with 0.6 M of NaCl, which led to an approximate fivefold increase of CgHog1‐PP signal. Treatment with sorbic acid, PAA, lactic acid, low pH, glycerol, H_2_O_2_, and, unexpectedly, also fluconazole led to significantly increased levels of CgHog1‐PP (Figure 3c).

### 2.4 CgHog1 is required for survival in macrophages but is dispensable for systemic infection

**Figure 4:**
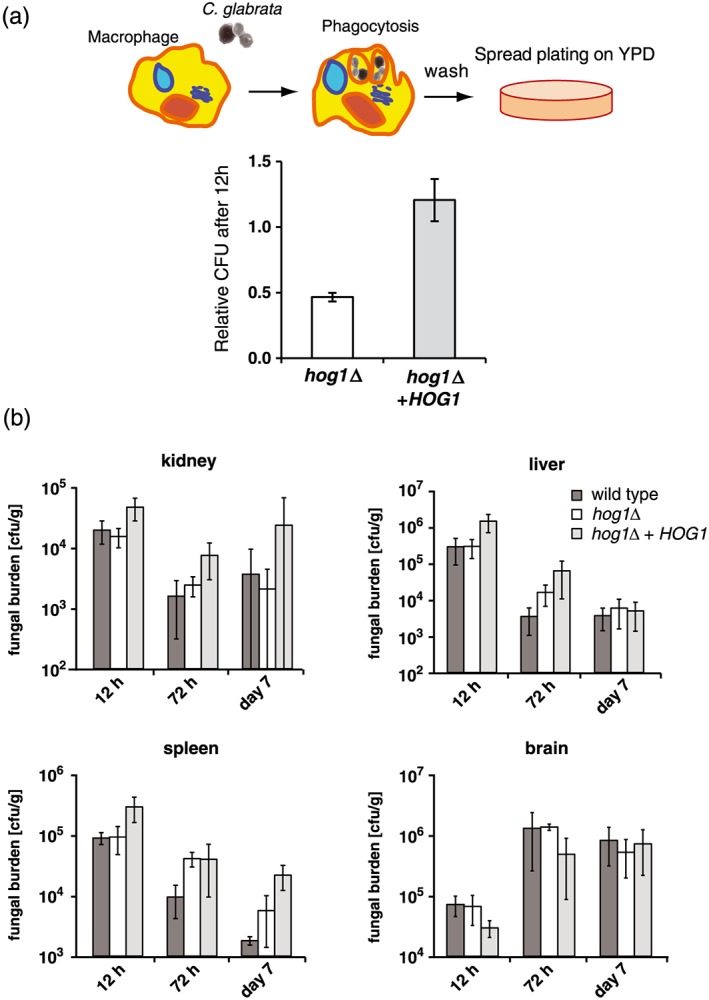
CgHog1 promotes survival in macrophages but not in a systemic mouse model (a) Cg*hog1*Δ as well as the reconstituted mutant strain were added to macrophages in a 1:2 ratio and nonphagocytosed yeast cells were removed after 45 min. Macrophage cells were lysed after 12 hr and *Candida glabrata* cells were spread on YPD plates; colonies were counted after incubation at 37°C for 2 days. (b) CFU count of C. glabrata wild type and *hog1*Δ cells after intravenous infection of CD1 mice. Five mice were infected per time point via the lateral tail vein with C. glabrata ranging from 2.5 × 10^4^ to 6.25 × 10^6^ CFU/g body weight. Samples of brain, kidneys, spleen, and liver were plated from mice after 12 hr, 72 hr, and 7 days of postinfection and CFU determined after 48 hr of incubation at 37°C. PBS mock‐infected animals served as controls

Macrophages and their ability to engulf and destroy pathogens are a part of the defence against many pathogens. We observed nuclear accumulation of CgHog1‐GFP after engulfment by murine bone marrow derived macrophages within the first hour, indicating activation of CgHog1 upon early phagocytosis. This activation was transient, as at the 12 hr time point the cytsolic CgHog1‐GFP fluorescence signal increased (Figure 3d), which could indicate CgHog1 inactivation. In addition, the survival of Cg*hog1*Δ mutant cells within macrophages was reduced after 12 hr of co‐cultivation (Figure 4a). To extend the analysis to a systemic infection model, we performed intravenous infections of CD1 mice with wild type, Cg*hog1*Δ and complemented mutant strains. CFU counts 12 hr, 72 hr, and 7 days of postinfection did not show significant differences in kidney, spleen, brain, and liver (Figure 4b). None of the mice succumbed to the fungal load in the course of our experiments (7 days). These results are in contrast to a previous observation that Cg*hog1* mutant cells have attenuated survival in a murine systemic infection model (Srivastava et al., 2015). We conclude from our data that the HOG pathway plays a role for defence against macrophages but no significant role in systemic infections, at least under the tested conditions in the CD1 mouse model.

### 2.5 The HOG pathway is essential for lactic acid resistance in clinical isolates of C. glabrata

**Figure 5:**
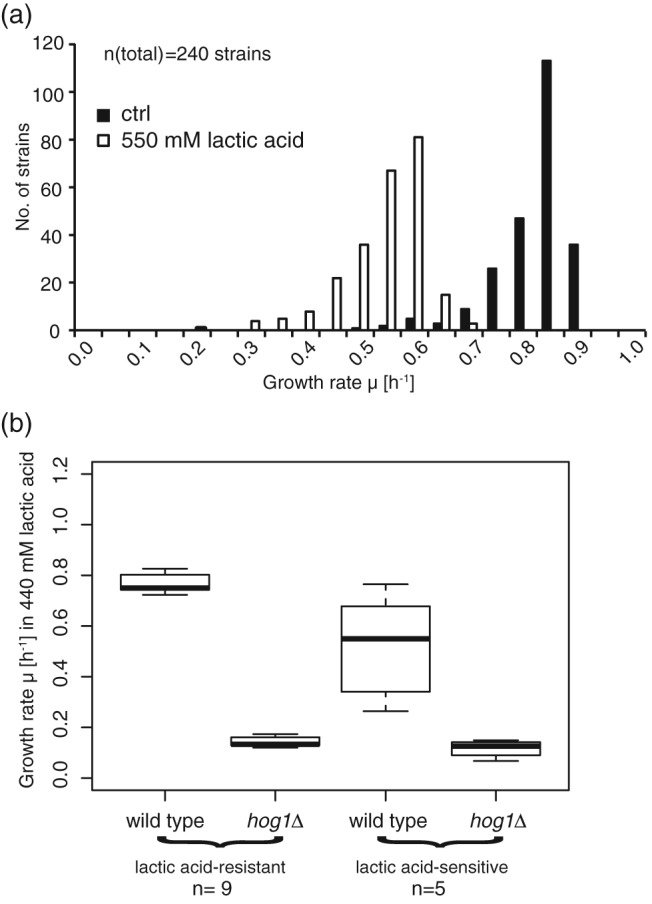
Hog1 is required for lactic acid tolerance in clinical *Candida glabrata* strains. (a) Population distribution of a strain collection of 240 clinical C. glabrata isolates obtained from a local federal hospital. Growth rates of all strains in the presence and absence of 550 mM of lactic acid adjusted to pH 4 were determined. (b) Growth rates of wild type and *hog1*∆ strains of clinical C. glabrata isolates previously showing a phenotype on lactic acid. Strains were cultivated on media containing 440 mM of lactic acid. Deletion of *HOG1* led to a drastic reduction of growth performance on lactic acid in both sensitive and resistant strains

To define other factors contributing to weak acid resistance, we identified lactic acid stress sensitive and resistant strains in a collection of 240 C. glabrata clinical isolates. This was done by quantitative growth analysis of the isolates when exposed to a high dose of lactic acid (550 mM; Figure 5a). To determine the contribution of CgHog1 for the resistance of these isolates, we deleted *HOG1* in nine resistant and five sensitive isolates and challenged them with different concentrations of lactic acid. Both resistant and sensitive isolates deleted for *HOG1* resulted in mutant strains, which had a very similar level of growth rate in the presence of lactic acid (Figure 5b and Figure S2A). This result indicates that the phenotypic variation among C. glabrata isolates is channelled through the HOG pathway and not a parallel pathway. A simple explanation for the different lactic acid susceptibilities in the clinical strains would be variation of stress sensing and signalling properties in these strains. We therefore investigated whether the phosphorylation status of CgHog1 under lactic acid stress was correlated with resistance in eight lactic acid resistant and eight sensitive C. glabrata strains (Figure S2B). Although there is a notable diversity in the CgHog1 phosphorylation pattern, the degree of the phosphorylation could not be correlated with growth rate on lactic acid. We assume that other, most likely downstream, components of the HOG pathway become limiting under lactic acid stress.

## 3 DISCUSSION

Being able to sense stress and to mount an appropriate response is vital, especially when simultaneously competing with other microbes in a changing environment (for review, see Brown et al., 2017). The HOG pathway is a central stress signalling pathway and has been explored in depth in S. cerevisiae (for reviews, see Saito et al., 2012, Brewster et al., 2014). In yeast, this pathway predominantly senses osmotic changes and is the major contributor of hyperosmotic stress resistance. Furthermore, ScHog1 is involved in sensing other stresses, that is, H_2_O_2_, citric acid, or acetic acid stress (Lee et al., 2017, Lawrence et al., 2004, Mollapour et al., 2006) and low pH (de Lucena et al., 2012, Claret et al., 2005). In C. albicans, Ca*HOG1* confers resistance to multiple stress types (Alonso‐Monge et al., 2003, Smith et al., 2004, Enjalbert et al., 2006, Brown et al., 2009) and is required for full virulence in a systemic mouse model (Alonso‐Monge et al., 1999). In C. glabrata, deletion of main constituents of the HOG pathway, such as *HOG1*, *PBS2*, *SSK2*, and *SHO1*, has been previously shown to induce susceptibility against not only osmotic stress but also weak organic acids (i.e., acetate or sorbate), heat stress, oxidative stress, and high external iron levels. This indicates that this pathway is responsible for a variety of stress responses in C. glabrata (Gregori et al., 2007, Kaloriti et al., 2012, Jandric et al., 2013, Srivastava et al., 2015).

Compared with *S. cerevisiae,* C. glabrata CgHog1 is activated by different stress types apart from osmotic stress. The results of our phenotypic screen concur with previous reports obtained with a C. glabrata *hog1*∆ mutant, which showed an increased susceptibility towards oxidative stress and low pH and no effect of BPS (Srivastava et al., 2015). However, we applied a quantitative approach by analysing additional cultivation parameters such as growth rate and lag phase. Interestingly, we did not observe a growth defect of HOG pathway mutants at 42°C in contrast to other reports (Srivastava et al., 2015, Gregori et al., 2007). This discrepancy, however, remained isolated and is possibly due to growth methods or strain background differences.

Patients defective in their macrophage or neutrophil response, e.g. patients suffering from neutropenia or those hospitalised in an ICU, are more likely to acquire a *Candida* infection and show a higher crude mortality than those with a fully functional innate immune response (Wisplinghoff et al., 2004). Thus, resistance to macrophage‐related killing is likely an important virulence trait of C. glabrata. *HOG1* confers resistance against low pH (<pH 4), nitrosative and oxidative stress, and could play a relevant role because macrophages kill their internalised prey with a combination of low pH and additional stress factors, such as reactive oxygen species and reactive nitrogen species. Our results indeed indicate activation of CgHog1 within macrophages and demonstrate its requirement for survival upon phagocytosis. However, we do not know whether CgHog1 has an active role in this confrontation or whether it simply provides sufficient basal stress resistance to survive the conditions within the partially matured phagosome. As C. glabrata evades killing by inhibiting phagosome acidification (Seider et al., 2011), it is possible that CgHog1 activation early after phagocytosis is involved in interference with phagosome maturation.

Macrophages use oxidative stress to attack engulfed microbes. C. glabrata strains have a relatively high intrinsic oxidative stress resistance (at least compared with S. cerevisiae), which seems independent of canonical antioxidant enzymes. The involvement of the HOG pathway in oxidative stress resistance of *C. glabrata* is unclear. Key enzymes of the ROS detoxification, Cg*CTA1*, Cg*SOD1*, and Cg*SOD2* are all dispensable in a murine infection model (Cuellar‐Cruz et al., 2008, Briones‐Martin‐del‐Campo et al., 2015). The catalase gene, Cg*CTA1*, is dispensable for survival within mouse macrophages as well (Roetzer et al., 2010). Both *C. glabrata* and S. cerevisiae are able to suppress ROS levels within macrophages to about the same extent due to active detoxification of ROS. However, the former is able to survive and replicate within the macrophage, whereas the latter is not (Seider et al., 2011). Previously, it has been reported that deletion of *HOG1* leads to impaired intracellular survival in C. glabrata when challenged with human THP‐1 macrophages (Srivastava et al., 2015). It was speculated that oxidative stress and high concentration of free iron within the macrophage cause the increased susceptibility of the mutant. We propose Cg*HOG1* to provide additional protection from macrophage killing. We show that, upon H_2_O_2_ challenge, Cg*HOG1* is required to restart the cell cycle as it reduced lag phase. A function for reducing the lag phase has also been reported for *S. cerevisiae* ScHog1 where it is required to restart the cell cycle after growth arrest (Escote et al., 2011). CgHog1 becomes phosphorylated but seems not to translocate into the nucleus. In fact, ScHog1 and CaHog1 translocation to the nucleus is not required for the activation of downstream events (Westfall et al., 2008, Lee et al., 2017, Day et al., 2017). Even though CgHog1 upon H_2_O_2_ challenge does not enrich in the nucleus, this does not exclude cycling between nucleus and cytosol. In any case, CgHog1 downstream targets are regulated, and it thus contributes to the rapid recovery promoting growth in the engulfed situation. We speculate that the combined action of low pH and oxidative stress within mammalian macrophage phagosomes necessitate the recalcitrance of C. glabrata during macrophage engulfment.

To further elucidate whether Cg*HOG1* plays a role in the pathogenicity of C. glabrata, we conducted an infection experiment in mice. Intravenous infection of mice is one of the most widely used *Candida* spp. virulence assays to date (Maccallum, 2012). Immunocompetent mice were described as a suitable model for testing the in vivo fitness of C. glabrata strains (Jacobsen et al., 2010, Brunke et al., 2015). Although in mice C. glabrata infections elicit only mild symptoms and a low induction of pro‐inflammatory cytokines, they can be recovered from host organs up to 28 days postinfection, even from immunocompetent mice (Brieland et al., 2001). Similar results were obtained when testing clinical strains of C. glabrata (Arendrup et al., 2002). We observed no altered fitness of the C. glabrata *hog1*Δ mutant in vivo. An earlier study in BALB/c mice (Srivastava et al., 2015) reported significant loss of recovered *hog1*Δ yeast cells 7 days postinfection. Our data collected from a time series show a rapid decline of fungal burden in the kidney, spleen, and liver within the first 3 days, except for the brain. We found no significant difference between wild type and *hog1*Δ mutant. The discrepancy to the Srivastava et al. study might originate from the different mouse and C. glabrata strains or technical differences. In any case, the results are suggesting that CgHog1 is not essential for fungal survival in a murine model.

Besides systemic infection, CgHog1 might be involved in commensal growth on mucosae and mucosal infections. On the human vaginal mucosa, C. glabrata must not only thrive under low pH but also compete with the vaginal microbiome, which consist predominantly of *Lactobacillus* spp. Vaginal dysbiosis and antibiotic treatment, which leads to depletion of *Lactobacillus* spp. in the vagina, are recognised as risk factors for VVC (Goncalves et al., 2016). *Lactobacillus* spp. secrete large amounts of lactic acid and a variety of other weak carboxylic acids thereby acidifying their environment and rendering it less hospitable for other microbes (Parolin et al., 2015, Larsen et al., 2001, Tachedjian et al., 2017). Their strategies encompass not only direct inhibition of competitive microbiota but also indirect inhibition through depletion of colonisation sites or competition for nutrients (Larsen et al., 2001). Interestingly, C. albicans responds to lactate with masking of β‐glucan, a key pathogen‐associated molecular pattern to reduce its visibility to the immune system and also indicating a long history of adaptation (Wheeler et al., 2008, Ballou et al., 2016).

Resistance to weak acids and low pH would allow C. glabrata to resist the *Lactobacillus*‐dominated vaginal microflora of a healthy host. Indeed, we found that Cg*HOG1* confers resistance to direct challenge with *Lactobacillus* spp. as well as to their late‐stationary phase supernatant. The growth‐inhibitory effect of *Lactobacillus* spp. on C. glabrata is likely caused by weak acids. We found lactic acid concentrations as high as 8 g/L (89 mM) in culture supernatants. The Cg*hog1*Δ mutants were severely inhibited at such concentrations on pH 4, which is similar to the vaginal pH. Our results from quantitative growth assays show that the inhibitory effect of organic acids relies mainly on the compound and not on the pH. Therefore, Cg*HOG1* confers resistance to associated stress factors of *Lactobacillus* spp. in co‐culture, indicating the relevance for persistence in vaginal milieu.

To define if the variation of lactic acid resistance is correlated with CgHog1 activation and phosphorylation, we selected lactic acid resistant and sensitive strains from a collection of clinical isolates. Deletion of *HOG1* in these selected isolates resulted in a uniform hypersensitive phenotype irrespective of the original phenotype. Therefore, the different phenotype is likely caused by differences downstream of CgHog1. Besides CgHog1, other components such as, for example, CgHaa1 might contribute to the variation of intrinsic resistance of isolates observed here. CgHaa1 regulates about 75% of all acetic acid induced genes (Bernardo et al., 2017) and deletion of Haa1 resulted in a growth phenotype on lactic acid and other weak organic acids. However, our results confirm Cg*HOG1* as a bottleneck in the complex regulative pattern of weak acid resistance.

In conclusion, the phenotypes conferred by Cg*HOG1* were associated with two important virulence factors: the ability to compete with vaginal *Lactobacillus* spp. and the ability to resist macrophage‐assisted killing. Cg*HOG1* also confers resistance to individual stresses such as oxidative, nitrosative, and weak acid stress enabling C. glabrata to compete with vaginal *Lactobacillus* spp. and to overcome the host's innate immune system. Finally, we report that the HOG pathway appears to be a bottleneck in the regulation network governing intrinsic weak acid resistance in C. glabrata. As a next step, the relevance of the HOG pathway for virulence might be assessed further, either in an improved in vivo model, such as a murine VVC model (Nash et al., 2016), or cell culture‐based models mimicking the vaginal microbiome. We propose that CgHog1 has a vital function in the host–commensal relation.

## 4 EXPERIMENTAL PROCEDURES

### 4.1 Strains, chemicals, and media

*Candida* strains were grown on YPD medium (1% yeast extract, 2% peptone, 2% glucose) and *Lactobacillus* spp. on MRS medium (DeMan Rogosa Sharpe). Standard incubation was performed at 37°C. Unless indicated otherwise, *Candida glabrata* strain BG14 (*ura3*Δ(*−285 + 932)::Tn903NeoR*) (Cormack & Falkow, 1999) was used as reference strain. The Cg*hog1*Δ mutant and the pHog1‐GFP plasmid were created as described previously (Jandric et al., 2013). The Cg*hog1*Δ mutant strain was confirmed by whole genome sequencing (30× coverage) to contain no additional mutations other than the intended knock out (data not shown). The Cg*HOG1* complemented Cg*hog1*Δ mutant (*hog1*Δ*HOG1*) was created by homologous recombination of a PCR‐amplified fragment containing the wild type Cg*HOG1* gene using Hog1_forward: 5′‐TTCACAGGATAGCAACG‐3′ and Hog1_reverse: 5′‐TGGATACAACCGGTAAC‐3′ primers. Revertants were selected on YPD agar containing 1.5 M of NaCl. From those, PCR positive clones for Cg*HOG1* lacking the NAT‐resistance cassette were selected. All *Lactobacillus* spp. were obtained from the general hospital of Vienna (AKH Vienna) and typed by 16S rRNA sequencing and MALDI‐TOF for species confirmation. Six different *Lactobacillus* isolates were selected for further experiments, one isolate each of *Lactobacillus fermentum*, *Lactobacillus casei*, *Lactobacillus crispatus*, 7*Lactobacillus paracasei*, *Lactobacillus gasseri*, and *Lactobacillus rhamnosus*. All strains obtained by the AKH were isolated from vaginal swabs of anonymous patients.

To test stress resistance, potassium sorbate (Sigma‐Aldrich/Merck KGaA, Darmstadt, Germany) was prepared from a 500 mM of stock and adjusted to pH 4. Lactic acid (Sigma‐Aldrich/Merck KGaA, Darmstadt, Germany) was diluted from an 80% *w*/*v* stock solution and adjusted to pH 4. Phenyl acetic acid (PAA) was prepared from a 200 g/L of stock (in 95% EtOH) and diluted with sterile distilled water. Fluconazole (Sigma‐Aldrich/Merck KGaA, Darmstadt, Germany) was prepared from a 330 g/L of stock solution (in 95% EtOH). Bathophenanthrolinedisulfonic acid (BPS; Sigma‐Aldrich/Merck KGaA, Darmstadt, Germany) was prepared from a 3.75 M of stock solution. Sodium nitrite was prepared from a 1 M of stock solution in water. To promote oxidative dissociation of nitrite into NO_x_ species, pH was adjusted to pH 4 with HCl. For growth inhibition tests (spottings), cell cultures grown until OD_600nm_ of 1 were diluted in 1:5 steps with sterile water; 3 μl of each dilution were spotted onto YPD agar plates containing the respective compound. For co‐cultivations with *Lactobacillus* spp., a lawn of the respective *Lactobacillus* spp. was grown aerobically on a petri dish containing MRS medium for 1 day at 37°C; C. glabrata strains from cultures grown o/n on MRS were then spotted onto the lawn and incubated at 37°C for at least 24 hr.

Clinical C. glabrata isolates were selected by growth phenotype on lactic acid and scored according to their growth rate. Resistant and sensitive isolates were randomly chosen from the Top and Bottom 10 percentile, and *hog1*∆ knockouts were created from those isolates. Strains used were according to our strain collection: CGC186, CGC138, CGC12, CGC184, CGC89, CGC29, CGC55, CGC150, CGC162, CGC214, CGC7, CGC193, CGC16, and CGC231. Briefly, a NATMX6‐cassette with flanking regions homologous to *HOG1* was created via PCR of genomic DNA from BG14 *hog1*∆ using primers Hog1_forward and Hog1_reverse and subsequently introduced into selected isolates by electroporation. Transformants were selected first on nourseothricin and then tested on 1 M of NaCl to confirm an osmo‐sensitive phenotype.

### 4.2 Drought resistance

In a 1:2 series in a round‐bottom 96 well plate, 10^7^ CFU of BG14 wild type and BG14 *hog1*Δ in triplicate were diluted in sterile water (Corning Inc., Corning, United States). The liquid was evaporated at 37°C at <1 atm. Plates were then incubated at 37°C. At the times indicated, a single plate was chosen, 200 μl of YPD added to each well and incubated for 48 hr at 37°C. Growth was evaluated visually. CFU reduction was estimated according to the lowest dilution that did not show growth after 48 hr.

### 4.3 Quantitative growth kinetics

Growth kinetics were quantitatively determined in an automated high throughput system in standard 96‐well flat bottom plates containing 200 μl of YPD in each well supplemented with the respective compound. Plates were prepared using a liquid handling robot and incubated for 24 hr at 37°C (Cytomat 2, ThermoFisher, Waltham, United States). OD_600nm_ was measured every 30 min using a fully automated set‐up (Synergy H1 reader, Biotek, Winooski, United States; Rack Runner 720, Hamilton Robotics, Martinsried, Germany). Growth curves were fitted using the GROFIT package in R (Kahm et al., 2010). Each experiment was run at least in triplicate on the same plate. The average growth obtained was then tested for significant deviation between wild type and mutant using Student's *t* test. For growth in the presence of *Lactobacillus spp*. supernatants, *Lactobacillus spp*. (L. rhamnosus, *L. paracasei*, and L. fermentum) were grown in YPD at 37°C under aerobic conditions until stationary phase was reached after about 48 hr. The supernatant was removed and filter‐sterilised using a 0.2 μm of polyethersulfone filter. HPLC analysis of the supernatant revealed high lactic acid contents of 6–8 g/L for all three supernatants. The supernatants were adjusted to pH 7, inoculated with wild type and *hog1*Δ mutant strains and incubated at 37°C. Growth analysis was performed as described above.

### 4.4 Fluorescence microscopy

Fluorescence microscopy was performed on a CellR microscope (Olympus, Tokyo, Japan). BG14 *hog1*Δ + pHog1‐GFP was inoculated in YPD from an o/n culture to OD_600nm_ 0.1 and incubated for ~5 hr at 37°C until OD_600nm_ ~0.8 was reached. Aliquots were treated with the respective stress agents for 15 min at 37°C. Heat‐stressed samples were incubated at 42°C in a block heater. For each condition, at least 40 individual cells from at least two independent experiments were analysed with ImageJ (Schindelin et al., 2015). For each cell, a nucleus and a cytosol area was selected and the mean intensity was calculated after background correction. The nucleus and cytosol (NC) ratios were subject to outlier removal using the Thompson Tau test. The final NC‐ratio values were tested for significance using the nonparametric Kruskal–Wallis test using the R environment. A *p* < 0.05 was assumed significant.

### 4.5 Western blotting

Twenty‐five millilitres of cultures were grown to an OD_600nm_ of 1 in YPD at 37°C and then treated accordingly for 15 min, except when indicated otherwise. Samples containing lactic acid, sorbic acid, nitrite, or PAA were adjusted to pH 4. For heat‐stress treatment, samples were incubated in YPD at 42°C for 15 min. Cells challenged with *Lactobacillus* supernatants were grown in YPD at 37°C to an OD_600nm_ of 1, harvested and resuspended in an equal volume of supernatant from a stationary culture of L. rhamnosus grown in YPD at 37°C for 3 days. To avoid effects caused by medium depletion, the supernatants were replenished with 1/10th volume of a 10× YPD stock before treatment. Total protein extracts were prepared using trichloracetic acid precipitation (Egner et al., 1996). CgHog1 phosphorylation levels were detected on blots using rabbit anti‐phospho‐p38 MAPK (Thr180/Tyr182; D3F9) mAb (Cell Signalling Technology No. 4511), detected with anti‐rabbit HRP‐conjugated antibody (Sigma‐Aldrich No. A 6154) and visualised by SuperSignal West Pico detection solution (Thermo Scientific). As a loading control, a rabbit antiserum raised against yeast phosphoglycerate kinase (Pgk1) was used (Kuchler et al., 1993). All signals were normalised to Pgk1 and to a reference protein extract of BG14 grown in YPD, which was run on each gel.

### 4.6 Macrophage assays

Primary bone marrow‐derived macrophages (BMDMs) were obtained from the femur bone marrow of 6‐ to 10‐week‐old C57Bl/6 mice housed under specific pathogen‐free conditions. Cells were cultivated in Dulbecco's Modified Eagle's medium supplemented with 10% fetal calf serum (FCS) in the presence of L cell‐derived CSF‐1 as described (Baccarini et al., 1985). For infection assays, BMDMs were seeded at 5 × 10^5^ cells per dish in 3.5 cm dishes containing medium without antibiotics. Log‐phase C. glabrata cells were washed with phosphate buffered saline (PBS) supplemented with 0.1% glucose and added to macrophages in a 4:1 ratio and incubated at 37°C. For microscopy, cells were fixed with 2% formaldehyde for 5 min. After washing with PBS, cells were incubated in 1% Triton X‐100 for 1 min. After another washing cycle, cells were dyed with Phalloidin Texas‐Red for 30 min. Coverslips were fixed to slides with Mowiol. For CFU assays, BMDMs were seeded at 2 × 10^5^ cells per dish. Exponentially growing C. glabrata cells were washed with PBS supplemented with 0.1% glucose and added to macrophages in a 1:2 ratio and incubated at 37°C. After 45 min, cells were washed three times with PBS to remove nonphagocytosed yeast cells, and fresh medium was added. At the indicated times, deionised water was added to lyse macrophage cells. C. glabrata cells were spread on YPD plates; colonies were counted after incubation at 37°C for 2 days.

### 4.7 Murine infection model

The infection was performed as described previously (Jacobsen et al., 2014). Briefly, C. glabrata cells from liquid YPD overnight cultures were washed twice with ice‐cold PBS and adjusted to the desired concentration of 5 × 10^8^ cells/ml in PBS. On Day 0, five female, 8–10 weeks old, CD1 mice per time point were infected via the lateral tail vein with 2.5 × 10^7^ CFU C. glabrata in 200 μl of PBS. The infection dose was confirmed by plating. After infection, the health status of the mice was examined twice a day by a veterinarian. Mice were humanely sacrificed 12 hr, 72 hr, and 7 days of postinfection. Immediately after euthanasia, brain, kidneys, spleen, and liver were removed aseptically, rinsed with sterile PBS, weighed, and placed in sterile PBS. Following homogenisation using an UltraThurrax, serial dilutions were prepared in PBS and plated on YPD for determination of CFU.

### 4.8 Ethics statement

All animal experiments were performed in accordance with the German animal protection law and were approved by the responsible Federal State authority (Thüringer Landesamt für Lebensmittelsicherheit und Verbraucherschutz) ethics committee (beratende Komission nach §15 Abs. 1 Tierschutzgesetz; permit no. 03‐003/12). The animals were cared for in accordance with the European Convention for the Protection of Vertebrate Animals Used for Experimental and Other Scientific Purposes.

## CONFLICTS OF INTEREST

The authors declare that no competing interests exist.

## Supporting information


**Figure S1:** **(A)** BG14 wild type (left panel) and BG14*hog1*Δ (right panel) were cultivated in YPD or in the presence of the indicated osmolyte in 96‐well format for 24 h at 37°C. Two different concentrations as indicated were tested for each stress condition. Maximal growth rate μ [h^−1^] was calculated with GROFIT. Each experiment was run at least in triplicate with three biological replicates. **(B)** Serial dilutions of wild type and mutant were distributed in 96‐well plates. After evaporation of the liquid, plates were incubated at 37°C for indicated numbers of days before survival was determined after 48 h incubation in liquid YPD. Log_10_‐reduction of growth is given for both strains. **(C)** Wild type and Cg*hog1*Δ were cultivated in 96‐well format in the presence of indicated concentrations of nitrite or H_2_O_2_ for 24 h at 37°C. OD_600nm_ was measured every 30 minutes in triplicate with three biological replicates on the same plate and lag phase was determined with GROFIT.

**Figure S2:** **(A)** Growth rates of selected clinical C. glabrata isolates on lactic acid. In strains previously identified as lactic acid‐resistant or lactic acid‐sensitive Cg*HOG1* was deleted. Wild type and mutant strains were cultivated on media containing different concentrations of lactic acid and the relative growth rate was determined. Lack of Cg*HOG1* leads to a comparable reduction of growth performance on lactic acid in both sensitive and resistant strains at all tested concentrations. (**B**) In a subset of 16 clinical C. glabrata isolates showing a phenotype on lactic acid (8 sensitive, full triangles and 8 resistant, open triangles) from Figure 5A, growth rate and CgHog1 phosphorylation in the presence of lactic acid was investigated. No significant correlation was found between the levels of phosphorylated CgHog1 at 100 mM lactic acid and the relative growth rate of the isolates at 100 mM lactic acid.
