# Supplementary figures and images for: Competition of Candida glabrata against Lactobacillus is Hog1 dependent
Source: Cell Microbiol. 2018 Sep 7;20(12):e12943. doi: 10.1111/cmi.12943 (PMC6283251; doi:10.1111/cmi.12943)

Figure S1

A

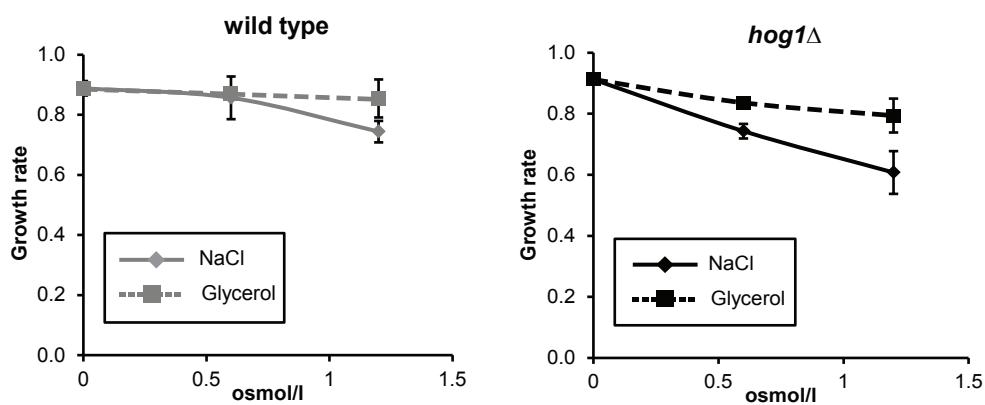

B

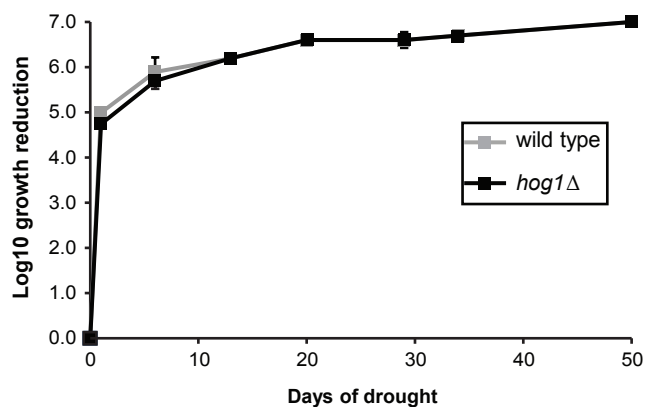

C

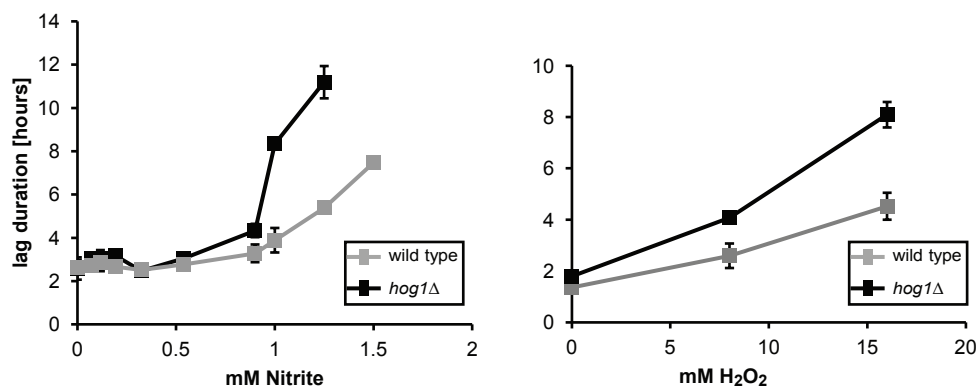

Supplement: Supplementary file 1 — Figure S1: (A) BG14 wild type (left panel) and BG14hog1Δ (right panel) were cultivated in YPD or in the presence of the indicated osmolyte in 96‐well format for 24 h at 37°C. Two different concentrations as indicated were tested for each stress condition. Maximal growth rate μ [h−1] was calculated with GROFIT. Each experiment was run at least in triplicate with three biological replicates. (B) Serial dilutions of wild type and mutant were distributed in 96‐well plates. After evaporation of the liquid, plates were incubated at 37°C for indicated numbers of days before survival was determined after 48 h incubation in liquid YPD. Log10‐reduction of growth is given for both strains. (C) Wild type and Cghog1Δ were cultivated in 96‐well format in the presence of indicated concentrations of nitrite or H2O2 for 24 h at 37°C. OD600nm was measured every 30 minutes in triplicate with three biological replicates on the same plate and lag phase was determined with GROFIT. [file CMI-20-na-s001.pdf]

Figure S2

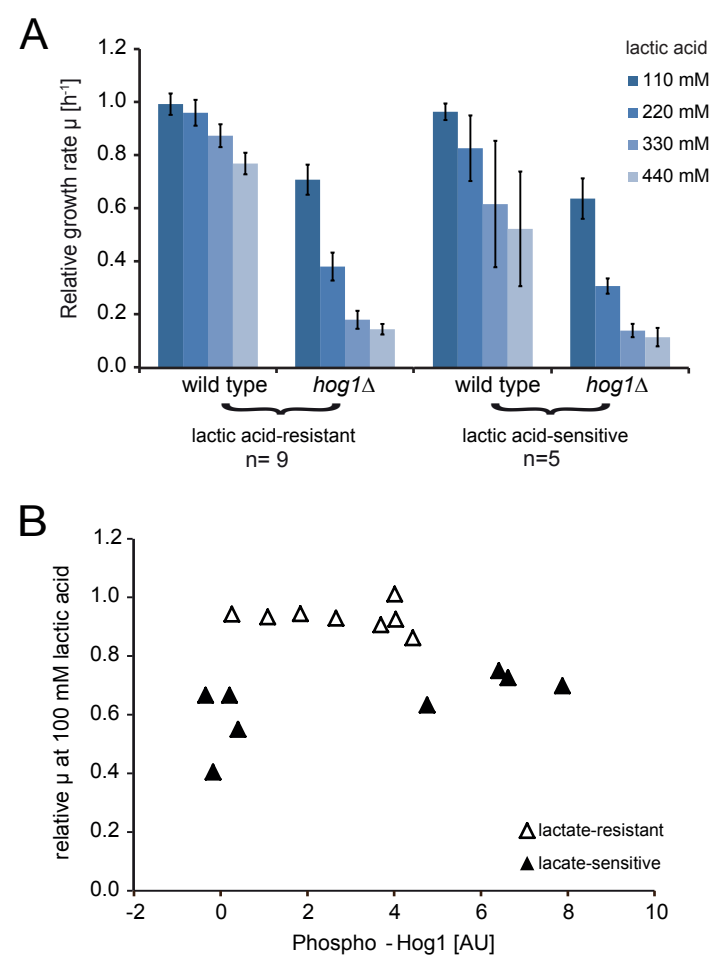

Supplement: Supplementary file 2 — Figure S2: (A) Growth rates of selected clinical C. glabrata isolates on lactic acid. In strains previously identified as lactic acid‐resistant or lactic acid‐sensitive CgHOG1 was deleted. Wild type and mutant strains were cultivated on media containing different concentrations of lactic acid and the relative growth rate was determined. Lack of CgHOG1 leads to a comparable reduction of growth performance on lactic acid in both sensitive and resistant strains at all tested concentrations. (B) In a subset of 16 clinical C. glabrata isolates showing a phenotype on lactic acid (8 sensitive, full triangles and 8 resistant, open triangles) from Figure 5A, growth rate and CgHog1 phosphorylation in the presence of lactic acid was investigated. No significant correlation was found between the levels of phosphorylated CgHog1 at 100 mM lactic acid and the relative growth rate of the isolates at 100 mM lactic acid. [file CMI-20-na-s002.pdf]
